# Attentional Requirements on Feature Search Are Modulated by Stimulus Properties

**DOI:** 10.1371/journal.pone.0053093

**Published:** 2013-01-07

**Authors:** Janne F. Ettwig, Adelbert W. Bronkhorst

**Affiliations:** 1 Vrije Universiteit, Amsterdam, The Netherlands; 2 TNO, Soesterberg, The Netherlands; University of California, Davis, United States of America

## Abstract

We report a series of dual-task experiments, in which a rapid serial visual presentation (RSVP) task was combined with a visual search task. Orientation, motion, and color were used as the defining target features in the search task. Lag between target onsets was manipulated and interference between the two tasks was quantified by measuring detection scores for the search task as a function of lag. While simultaneous performance of an orientation detection task with an RSVP letter identification task resulted in a performance decrease for lags up to 320 ms, no such decrease was detected for highly salient motion- and color-defined targets. Subsequently, detectability of the motion and color feature was matched to that of the orientation-feature resulting in the reintroduction of a (smaller) performance decrease, but only during simultaneous performance (lag 0 ms). The results suggest that there are two causes for the impaired search performance occurring when a feature search task is combined with an RSVP task. The first is short-lasting interference probably due to attentional competition; the second, which plays a role only when targets for both tasks share features, is interference that may be attributed to a central processing bottleneck.

## Introduction

We all know that sometimes it is difficult be spotted in a crowd, for example, when meeting friends in a bar. One way to make this search easier is by dressing in distinct colors that make one stand out. However, if a bright red jacket is not your taste, waving your arm may also work. These phenomena have been explored extensively in laboratory studies, which demonstrate that unique motion and unique color can indeed attract attention and thus greatly facilitate searching a target among distractors (e.g. [1]–[10]). It is however not entirely clear whether search for unique targets is equally efficient in dual-task conditions.

The two-stage theory is one of the dominant models for visual processing (e.g., [11]–[14]). According to this theory visual processing is divided into a preattentive *parallel* stage and an attentive *serial* stage (for a recent review, see [15]). In the preattentive stage, attention is guided by the salience of elements in the visual field; these elements are then further analyzed in the attentive state (e.g., [11], [13], [16]).

In visual search displays, attention is often attracted by unique attributes like color, motion, size, luminance, and flicker (e.g., [1]–[7], [9], [10]). Features that attract attention are called salient; they stand out relative to their neighbors. For example, searching for a red dot among green dots is easy, no matter how many green dots surround it. In other words, in these cases the target seems to pop out from the display. This leads to search times that are independent of the number of elements in the display (set size), as indicated by search slopes that are basically flat. The abovementioned theory proposes that targets defined by these attributes can be detected at the preattentive stage of processing, which means that no focal attention would be required (e.g., [12], [13]).

However, Joseph, Chun and Nakayama [17] argue that focal attention is indispensable to process any kind of stimulus. To demonstrate this, they employed a dual-task paradigm to manipulate attention during visual search. In the first task participants observed a rapid serial visual presentation of letters (RSVP; [18]) in which they identify one uniquely colored letter (T1). The letters are presented at the same location and in quick succession, which makes this task highly demanding and resource consuming [17], [19]. The second task was a visual detection task, wherein a uniquely oriented Gabor patch (T2) had to be detected among uniformly oriented distractor patches. When this orientation-based detection task was performed in isolation, a uniquely oriented patch gave a clear pop-out, as shown by flat search slopes for increasing set sizes. In the dual-task condition, time between T1 and T2-search display presentation was manipulated to test the influence of T1 processing on T2 detection. When participants are asked to perform both tasks, a drop in T2 detection performance was shown for lags up to 400 ms between targets. These results suggest that successful processing of an orientation-defined target is hindered when attention is engaged elsewhere within this temporal window of 400 ms. This is unexpected, because when combining two visual discrimination tasks they typically only interfere when both have steep search slopes (e.g., [13], [16], [20]–[24]). The authors interpret these results as evidence that attention is needed for the detection of any type of visual stimulus.

Others came to similar conclusions. For example, Theeuwes, Van der Burg and Belopolsky [25] used a visual search task with identity priming across trials to show that detecting the presence or absence of a single, colored “pop-out” target requires spatial attention. These findings are consistent with several studies demonstrating that even the simplest possible feature detection requires spatial attention [9], [24], [26]–[28]. The underlying notion is that postselective processing is obligatory for a detection response and occurs automatically even in tasks in which identifying the target is not necessary. Using a different approach, Theeuwes, Kramer and Atchley [29] showed in a location cueing task that the allocation of attention in visual space has a large effect on the speed with which participants can detect the presence of a feature singleton. Spatial attention can produce reaction time benefits or costs depending on its relative location to a basic-feature target.

However, there is also an alternative explanation for the decrease in performance in a dual-task condition as found by Joseph et al. [17]: insufficient T2 saliency. Processing some type of features seems to require more attention than others (e.g., [13], [16], [20]–[24], [30]), yet processing can also be very rapid, making it almost undetectable [1], [3], [31]. Highly salient features, like color or motion, can be processed even when attention is engaged elsewhere [1], [3], [31], [32]. Chua [33] and Shih and Reeves [34] explored effects of salience in dual-task conditions. They used an attentional blink paradigm showing the characteristic drop in T2 identification performance in the baseline condition, when T1 and T2 were equally salient. After increasing T2 luminance or making its color unique, the performance decrease disappeared. Increasing target saliency can thus boost detection in conditions where target detection is initially difficult.

A further, complementary, explanation for the results found by Joseph et al.’s [17] may be that there is feature-specific interference occurring between the two tasks used in their experiment. Previous research suggests that different stimulus dimensions are processed by separate subsystems, possibly with differentiated attentional capacities. Therefore, when two targets of the same dimension are to be processed simultaneously this may result in interference [35]–[39]. In line with this account, Ho [32] showed, using a dual-task paradigm, that two simultaneous second-order motion tasks interfere with each other, while the combination of a second-order motion task and an RSVP task does not. In the paradigm used by Joseph et al. [17], letter discrimination and the detection of an orientation-feature both require processing of orientation information, as a letter is made up by lines in different orientations [40]. This could explain why a performance reduction is found when these tasked are combined. However, results from other studies indicate that such dual-task interference may only occur in specific cases. Duncan and colleagues compared single- with dual-task performance on feature judgment tasks for either similar or dissimilar features [6], [7], [10]. Discriminations had to be performed on either one or two targets, and had to be judged on form, color and motion, amongst others. Critically, no great difference in interference was found for combinations of similar or dissimilar tasks leading the authors to conclude that attentional capacities used for processing different types of features are undifferentiated.

Because it is not clear to what degree the results of Joseph et al. [17] depend on the specific combination of features used, we investigate in the current study how a search tasks with different types of target-defining features interacts with an RSVP task. Participants were asked to identify a white letter (T1) in a black-letter stream, while also detecting a target in a visual search display (T2) surrounding the letter-stream. Time between targets was manipulated to examine dual-task interference as a function of lag. After replication of Joseph et al.’s [17] results with orientation-defined targets in Experiment 1, two different T2 features are tested in the subsequent experiments: motion in Experiment 2a and color in Experiment 2b. Experiment 3a and 3b are in essence replications of Experiments 2a and 2b, but with reduced T2 saliency. A staircase procedure was included to equate detectability of the motion and color targets in the single-task condition to that of the orientation target in Experiment 1. Our expectation was to find at most a small performance decrease in the dual-task conditions in Experiments 2a and 2b, not only because motion and color are highly salient features, but also because no interference is expected between the processing of these features and the processing of the letter in the RSVP stream [7], [35]–[38], [41]. Since we attempted to remove effects of saliency differences in Experiments 3a and 3b, the results of these experiments should indicate whether the latter factor – interference during processing – indeed plays a role.

## Experiment 1– Orientation

### Methods

#### Ethics statement

The present and all following experiments, including the consent procedure, were approved by the ethics board of the Faculty of Psychology and Education (VCWE) and conducted according to the principles of the Declaration of Helsinki. Participants received information about the study and their rights and gave a written informed consent. The study was not associated with any risks for participants (it was non-invasive) and all data obtained during this study was analyzed anonymously.

#### Participants

Fourteen students (9 female; one left-handed; mean age 21 years; age range 18 to 30 years) participated for course credits or money. All had normal or corrected-to-normal vision.

#### Stimuli & apparatus

Participants were seated in a dimly lit room looking at the visual stimuli that were displayed on a 19-inch CRT monitor with a 120 Hz refresh rate, on a gray background (25 cd/m^2^). The participants viewed the monitor binocularly from a 57 cm-distance with their head placed in a headrest.

**Figure 1:**
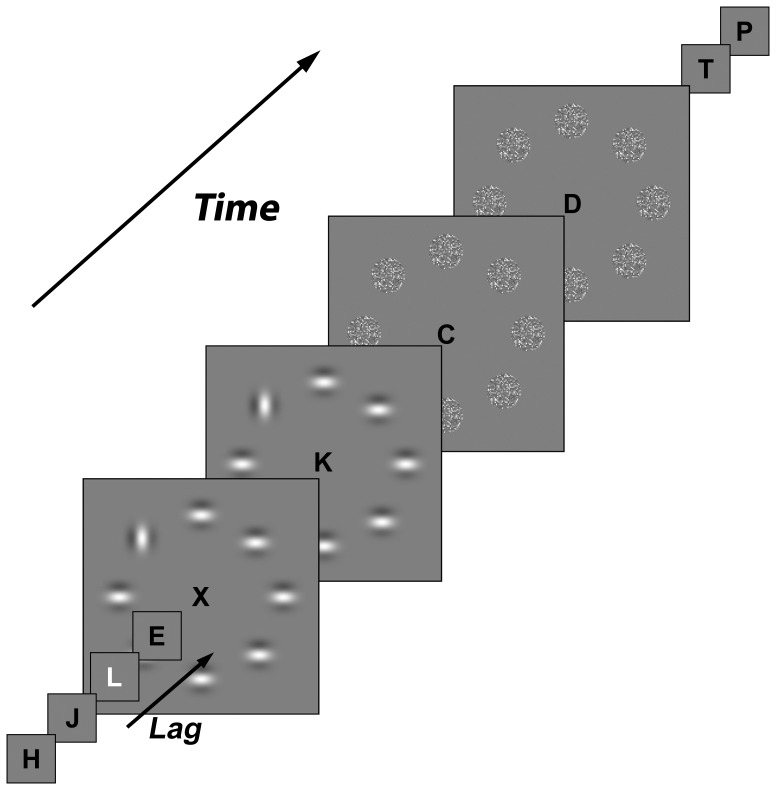
Schematic overview of trial timeline from left to right. Letters were presented for 33 ms alternated by 50-ms blanks. The white target letter (T1) was followed by a lag of 0, 160, 320, or 640 ms, after which a ring of Gabor patches (containing T2) was presented. The ring of Gabor patches and mask screen were on screen for a duration of 160 ms each. Letter size is enhanced for illustration purposes.

In this dual-task experiment, the first task comprised the identification of a white target letter (T1) in a Rapid Serial Visual Presentation (RSVP) stream of black letters. The RSVP stream consisted of randomly (without replacement) chosen black, uppercase, letters, “X” excluded (0 cd/m^2^; 36 arcmin tall; font Geneva). T1 was a white, uppercase, letter (96.5 cd/m^2^; 36 arcmin tall) randomly chosen from the same set as the non-targets and could be displayed at the sixth to eleventh position in the RSVP stream. T1 was present in every trial and always followed by 14 letters. Each letter was presented for 33 ms followed by a 50-ms blank interval, see Figure 1. The second task consisted of detecting a differently oriented Gabor patch (T2) in-between homogenously oriented distractor patches. The Gabor patches were constructed with a Gaussian envelope of 50% peak contrast and a standard deviation of 22 arcmin. They had a cosine modulation of 110 arcmin wavelength and were either horizontally or vertically oriented. The Gabor patches were regularly spaced on an imaginary circle at 5.3° eccentricity and were always presented after a lag following T1. A target in the form of a Gabor patch that differed in orientation from the distractors was present in 50% of the trials. For an overview over the experiment timeline, see Figure 1.

#### Procedure & design

Participants either performed a single-task condition in which they detected T2 but ignored T1, or a dual-task condition in which they both identified T1 and detected T2.

The participants were instructed to maintain fixation on the RSVP stream at all times during the experiment. For practice, participants first viewed screenshots of the search display and then performed 10 trials of each condition first with and then without feedback. During the experiment itself no feedback was given. Each trial started with the presentation of a fixation dot for 500 ms followed by a 500-ms blank screen after which the RSVP stream started. At one of four different lags (0, 160, 320 or 640 ms) after T1-onset, the ring of Gabor patches was presented. Note that a lag of 0 ms implies simultaneous onsets of both T1 and the ring of Gabor patches. The Gabor patches were presented for 160 ms and were followed by a mask for the same duration while the RSVP stream continued, as illustrated in Figure 1. The mask consisted of a pixilated and scrambled Gabor patch. After each trial, participants gave an unspeeded response to T2 detection, pressing the “J” key when they did detect T2 or the “N” key when they did not. In the dual-task condition, they additionally responded to T1 by pressing the corresponding letter on a QWERTY keyboard.

The experiment consisted of 8 alternating blocks of single- and dual-task conditions, each comprising 48 trials with condition order counterbalanced across participants. Within the blocks T1 position, T2 presence and location, and lags were randomized across trials. Orientation of the Gabor patches and T1 identity were randomized. The experiment took approximately 45 minutes to complete.

### Results and Discussion

In all experiments, performance data for T2 detection is analyzed given correct T1 identification. T2 accuracy is based on correct performance, i.e. based on both trials in which T2 was correctly detected and trials in which T2 was correctly rejected.

We should note that, because only trials with a correct T1 were used to determine T2 performance, the number of available trials varies as a function of lag. In particular at lag 0 ms, 10–15% less trials were available than at other lags. Also in the following experiments, reductions up to 18% occur, also at lag 0 ms. This could have as effect that T2 results at lag 0 ms are less reliable than at other lags. Such a trend is however not reflected in the dependence of the size of the confidence interval on lag, and we also did not expect this given the total number of available trials for the lag 0 ms conditions was always large (more than 34 per participant).

#### T1 identification

**Figure 2:**
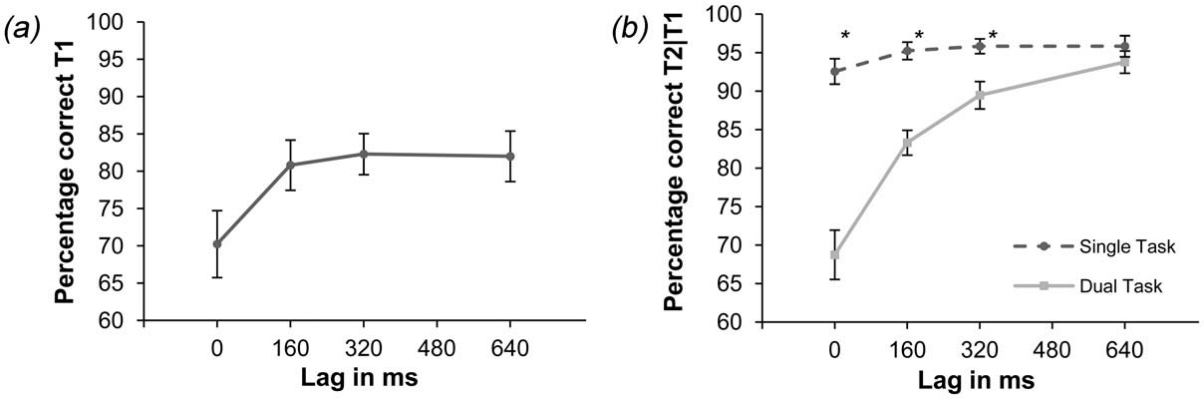
Results of Experiment 1. Panel (a) Percentage correctly identified letters in the dual-task condition. T1-identification in the dual-task condition was significantly lower for lag 0 ms than for all other lags. Panel (b) T2 detection performance in percentage correct in the single- and dual-task conditions. Dual-task results are based on trials with correctly identified letters. T2-detection in the dual-task condition is significantly lower for lags 0 ms, 160 ms and 320 ms than in the single-task condition. Stars mark significant differences between single- and dual-task condition per lag (p<0.05). Note that in this figure chance level is 50%. Error bars reflect 95% confidence intervals in both panels.

Looking at the letter identification data (see Figure 2A), a performance decrease at lag 0 ms is visible, and performance averaged at 77.78% correctly identified letters over all lags.

To evaluate this statistically, six Bonferroni-corrected paired-samples t-tests (*α* = 0.008) were performed. Results show that T1 identification in the dual-task condition was significantly lower for lag 0 ms than for all other lags (*p*<0.007).

#### T2 detection

The data for T2 detection (see Figure 2B) indicate that performance in the single-task condition was better than in the dual-task condition, and markedly so in the earlier lags. Performance averaged at 94.87% correct for the single-task condition versus 83.3% correct in the dual-task condition.

An analysis of variance (ANOVA) on this data revealed an effect of condition [*F*(1, 13) = 49.93, *MSE* = 3416.52, *p*<0.001] and lag [*F*(2,28) = 31. 5, *MSE* = 1086.09, *p*<0.001], as well as an interaction between condition and lag [*F*(2, 28) = 24.64, *MSE* = 622.32, *p*<0.001]. Four Bonferroni-corrected paired-samples t-tests (*α* = 0.013) showed that T2-detection in the single condition differed for lags 0, 160 and 320 ms from the dual condition (*p*<0.001).

Results show the same performance decline during the shorter lags of the dual-task condition, as found by Joseph et al. [17]. While performance on the single task remains constant over lags, simultaneous execution of the two tasks leads to interference between the two tasks at all lags except the longest. In order to determine whether this effect is feature specific, we replaced the orientation-feature with different types of features in the next two experiments.

## Experiments 2a & 2b – Motion and Color with High Salience

In Experiment 2a and b, we use motion and color, respectively, as the target-defining features in the T2 detection task. Combination of a motion detection task and an RSVP task was earlier shown to be possible without performance costs [32]. Therefore, when replacing the orientation T2 with a motion-defined T2, no performance decrease in the dual-task condition should be expected. Likewise, detection of a color-defined T2 should not be impaired by simultaneous T1 identification, consistent with Braun [1], [2] and Braun and Julesz [3], who concluded that color discrimination is essentially free of attentional cost.

### Methods

#### Participants

Sixteen students (2 male; one left-handed; mean age 19.9 years, age range 18 to 24 years) were recruited for Experiment 2a and sixteen different students (6 male; all right-handed; mean age 21.2 years, age range 18 to 29 years) for Experiment 2b. The students received course credits or money for participation and all had normal or corrected-to-normal vision.

#### Stimuli, apparatus & design

Apart from the following changes, Experiment 2a was the same as Experiment 1. In the Gabor patch search display the orientation-defined T2 was replaced by a motion-defined T2. T2 had the same orientation as the distractors and the Gaussian envelope remained fixed in position. To animate the target patch, the cosine-wave decreased or increased in phase with each presentation frame, thus creating left/right or up/down movement, depending on its orientation. During the presentation of the Gabor patch search display (160 ms) one full phase-cycle was completed before presenting the mask. Distractor/Target orientation, motion direction and target location were randomized. Experiment 2b differed in the following ways from Experiment 1. In the Gabor patch search display the orientation-defined T2 was replaced by a color-defined T2. Participants detected a unique color patch in-between homogenously colored distractor patches. The stimuli no longer had a Gaussian overlay, but were uniformly colored. The target and distractors were either red (255, 0, 0 in RGB values) or green (0, 152, 0) color patches and had an equal luminance of 25 cd/m2 as to the grey (75, 75, 75) background they were displayed on. Distractor/Target color was randomized. Furthermore, the experiment was displayed on a 23-inch LCD monitor with a 60 Hz refresh rate which allowed better color calibration than the CRT monitor used for the other experiments.

### Results and Discussion

#### T1 identification

**Figure 3:**
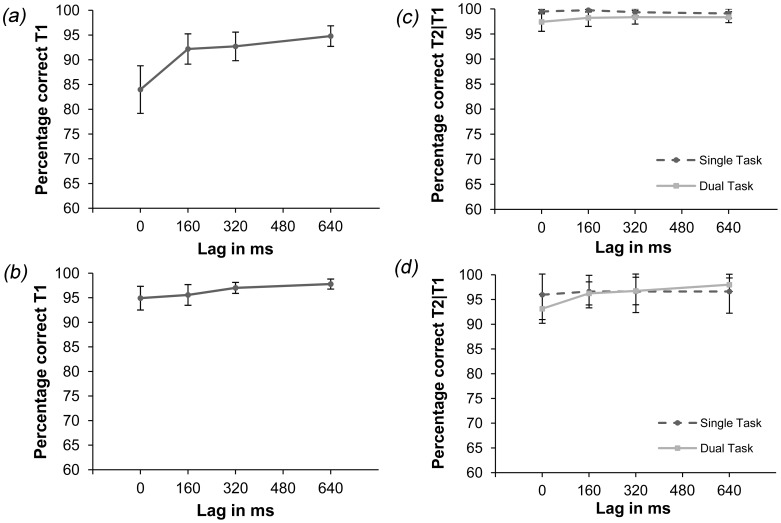
Results of Experiment 2. Panel (a) Percentage correctly identified letters in the dual-task condition of Experiment 2a. Significantly less letters are correctly identified for lag 0 ms compared to lags 320 ms and 640 ms. Panel (b) Percentage correctly identified letters in the dual-task condition of Experiment 2b. T1-identification was constant over all lags. Panel (c) T2 detection performance in percentage correct in the single- and dual-task conditions of Experiment 2a. Scores for the dual-task condition are for correct letter identification. No difference between conditions was detected. Note that in this figure chance level is 50%. Panel (d) T2-detection performance in percentage correct in the single- and dual-task conditions of Experiment 2b. Scores for the dual-task condition are for correct letter identification. No difference between conditions was detected, however performance for lag 0 ms was lower compared to lag 640 ms. Note that in this figure chance level is 50%. Error bars reflect 95% confidence intervals in every panel.

The data for letter identification in Experiment 2a (see Figure 3A) show a performance decrease during lag 0 ms. Averaged over all lags 90.92% of all the letters were correctly identified. In Experiment 2b (see Figure 3B), letter-identification performance was stable over time and averaged at 96.3% correctly identified letters over all lags.

Six Bonferroni-corrected paired-sample t-tests (*α* = 0.008) were used to analyze the potential performance difference between lags. For Experiment 2a T1 identification differed for lag 0 ms from lags 320 and 640 ms (*p*<0.002 for both comparisons, with less letters correctly identified at lag 0 ms). For Experiment 2b no such difference was found.

#### T2 detection

In Experiment 2a the target-defining feature was motion. T2 detection in the single-task condition appears not to differ from the dual-task condition, even at the earlier lags (see Figure 3C). Correct detection of the moving Gabor patch averaged at 99.4% correct for the single-task condition versus 98% correct in the dual-task condition. To examine T2-detection performance, a 2×4 ANOVA with condition (single/dual task) and lag (0, 160, 320 and 640 ms) as factors was performed. As expected, no effect was found. Following the line of reasoning of Joseph et al. [17] not enough resources should have been available for T2 processing, while attention was focused on and depleted by T1 processing. However, no performance decline was detected, consistent with the results of Ho [32] and Braun and colleagues [1]–[3]. This suggests that either our or Joseph et al.’s [17] findings are feature specific which prompts the question whether interference such as shown by Joseph et al. [17] can be demonstrated for another target-defining feature.

In Experiment 2 b the target-defining feature was color. Performance in the single- and dual-task conditions did not differ (see Figure 3C). The percentage correctly identified T2’s in the single-task condition averaged at 96.5%, for the dual-task condition this was 96%, given correct T1 identification. A 2×4 ANOVA with the factors condition (single/dual task) and lag (0, 160, 320 and 640 ms) was performed on the data in Figure 3C. An effect for lag [*F*(3, 45) = 4.98, *MSE* = 9.1, *p*<0.005] but no interaction between condition and lag was found [*F*(3, 45) = 2.54, *MSE* = 9.9, *p*<0.069]. Six Bonferroni-corrected paired-samples t-tests (*α* = 0.008) showed that T2-detection at lag 0 ms differed from that at lag 640 ms (*p*<0.003). Also, a rather high average performance level was observed, similar to that in Experiment 2a.

Interestingly in Experiment 2b also no significant performance decrease was found in the dual-task condition for T2, consistent with Braun [1], [2] and Braun and Julesz [3]. A color feature is processed in a separate subsystem from an orientation-feature, possibly leading to less interference in a dual-task setting. However, an alternative explanation is also possible. As discussed in the Introduction, target salience can boost performance for T2 in both the single- and the dual-task conditions. As a result, single-task performance in Experiments 2a and 2b approached maximum performance, such that dual-task interference might well have been obscured by a ceiling effect. In Experiments 3a and 3b we therefore increase task difficulty in order to equate single-task performance for all three target types.

## Experiments 3a & 3b – Motion and Color with Reduced Salience

In Experiment 3a and 3b we again use motion and color respectively as the T2 target-defining feature in the detection task. However, to rule out the possibility of a confound due to saliency differences, and to prevent any ceiling effect, we matched single-task performance for motion detection to that for orientation detection in Experiment 1 using a staircase procedure.

### Methods

#### Participants

Fourteen students (7 female; all right-handed; mean age 22.4 years, age range 18 to 32 years) were recruited for Experiment 3a and fourteen different students (7 male; one left-handed; mean age 22.3 years, age range 19 to 27 years) for Experiment 3b. They received course credits or money in exchange and all had normal or corrected-to-normal vision.

#### Stimuli, apparatus & design

Apart from the following changes, Experiments 3a and b were the same as Experiment 2a and b. Instead of 4 different lags, only 3 lags (0, 160 and 640 ms) were tested to limit experiment duration. Furthermore, the experiments were preceded by a staircase procedure, to be completed after the practice trials, designed to match the individual single-task performance to the average single-task performance found in Experiment 1. This was realized by employing a 1-up 13-down procedure designed to attain 94.8% correct answers (compared to 94.87% in Experiment 1). The number of required correct trials n for obtaining the proportion correct p in the up-down procedure was calculated using the formula p = 100*(0.5^∧^(1/n)), derived from Levitt [42]. The staircase procedure started at maximum detectability, i.e. a contrast of 100% for Experiment 3a and maximal color difference of red/brown for Experiment 3b.

In the staircase preceding Experiment 3a, the contrast of all the stimuli in the T2 search screen was decreased with steps of 1% after every sequence of 13 correctly answered trials, and increased with 1% after one trial was answered incorrectly. Until the first error was made, 3 instead of 13 correct responses were required for a contrast reduction, to speed up the procedure. When approaching the (previously piloted) range of intended performance levels (starting at 4%), the step size was decreased to around the just noticeable difference level of 0.1% [43]. In the staircase preceding Experiment 3b the colors were gradually varied from brownish to red tinges during the staircase procedure. This was achieved by decreasing the red-value of the RGB color value and adding green until near equal luminance (*stdev* = 0.029) of the original red color (RGB-value: 255, 0, 0; luminance 17 cd/m2) was reached. In this manner 46 colors were generated. For the complete range of colors and luminance, see Table S1 of the supporting information. Colors were defined according to the sRGB (standard RGB) color space, defined by the International Electrotechnical Commission (IEC) as IEC 61966-2-1 (1999) [44]. Target color, distractor/target color difference and target location were randomized. The step size in color difference Delta E was calculated according to the standard CIE76-1988. Delta E, a measure of subjective color difference, averaged at 0.83 (*stdev* = 0.11) between each step and was well below the just noticeable difference Delta E of approximately 2.3 [45]. The color difference between target and distractors, expressed in Delta E, had a (clearly discernible) value of 35 at the start of the staircase, and was decreased by 1 unit after every sequence of 13 correctly answered trials, and increased by 1 when one trial was answered incorrectly. Until the first error was made only 3 correct answers were required for a decrease of Delta E in order to speed up the procedure.

For both experiments the staircase procedure was terminated after eight reversals [46] and the averaged contrast or Delta E of the last 6 reversals were calculated. This contrast or Delta E were then used for the subject during the subsequent experiment. The staircase procedure lasted 15 to 20 minutes, depending on performance. Experiment 3a consisted of 8 blocks, each comprising 36 trials. Each block had an equal number of trials for each contrast, presented in a random order. The experiment took approximately 50 minutes to complete. Experiment 3b consisted of 4 blocks, each comprising 120 trials and took approximately 45 minutes to complete.

### Results and Discussion

Subjects who, during the experiment, achieved scores of more than one standard deviation (calculated over all participants in the present experiment) below the target score of 94.8% were excluded from the analysis. In total 4 subjects were excluded: 2 in Experiment 3a and 2 in Experiment 3b. Their scores were 65%, 85%, 81% and 85%, respectively.

#### T1 identification

**Figure 4:**
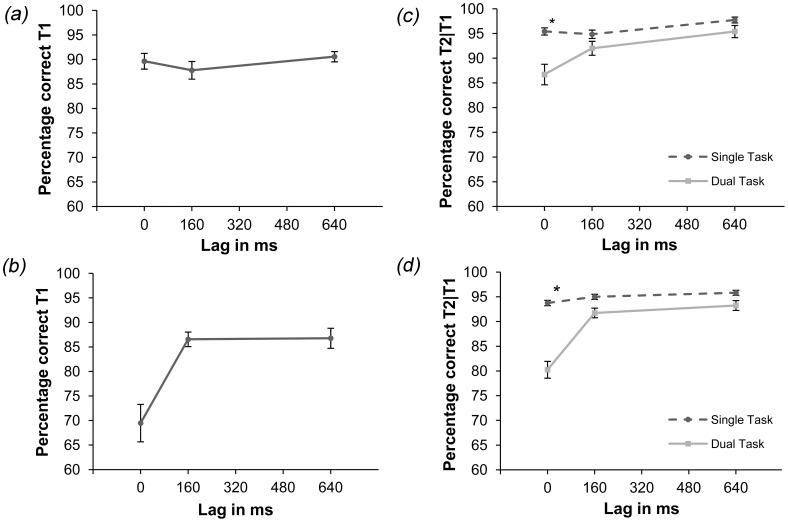
Results of Experiment 3. Panel (a) Percentage correctly identified letters in the dual-task condition of Experiment 3a. T1-identification was constant over all lags. Panel (b) Percentage correctly identified letters in the dual-task condition of Experiment 3b. Significantly less letters are correctly identified for lag 0 ms compared to lags 160 ms and 640 ms. Panel (c) T2-detection performance in percentage correct in the single- and dual-task conditions of Experiment 3a. Scores for the dual-task condition are for correct letter identification. Performance in the dual-task condition was significantly lower than in the single-task condition at lag 0 ms. Stars mark significant differences between single- and dual-task condition per lag (p<0.05). Note that in this figure chance level is 50%. Panel (d) T2-detection performance in percentage correct in the single- and dual-task conditions of Experiment 3b. Scores for the dual-task condition are for correct letter identification. Performance in the dual-task condition was significantly lower than in the single-task condition at lag 0 ms. Stars mark significant differences between single- and dual-task condition per lag (p<0.05). Note that in this figure chance level is 50%. Error bars reflect 95% confidence intervals in every panel.

In Experiment 3a, letter-identification performance was stable over time, as shown in Figure 4A. Averaged over all lags 89.3% of all the letters were correctly identified. In Experiment 3b, letter identification showed a noticeable performance decrease at lag 0 ms (see Figure 4B). Averaged over all lags, performance was 80.9%.

Four Bonferroni-corrected paired-sample t-tests (*α* = 0.0125) analyzed the potential performance difference between lags. In Experiment 3a T1 identification did not differ significantly over lags. In Experiment 3b T1-identification performance in lag 0 ms differed from lags 160 and 640 ms (*p<*0.001), with participants scoring significantly lower at 0 ms than at the other lags. Furthermore, the differences in letter task performance between experiments were also evaluated with two Bonferroni-corrected (*α* = 0.025) independent t-tests. No significant difference could be detected between Experiments 3b and 1 or between Experiments 3a and 3b.

#### T2 detection

The average patch-contrast that resulted from the staircase procedure was 2.35%. The data of Experiment 3a for T2 detection indicate that performance in the single-task condition was better than in the dual-task condition, most notably in the earliest lag (see Figure 4C). Correct detection of the moving Gabor patches averaged at 96% correct for the single-task condition versus 91.4% correct in the dual-task condition. T2-detection performance was examined with an 2×3 ANOVA with condition (single/dual task) and lag (0, 160 and 640 ms) as factors. Effects for condition [*F*(1,11) = 9.13, *MSE* = 42.32, *p*<0.012] and lag [*F*(2,22) = 5.78, *MSE* = 31.66, *p*<0.01], and an interaction effect between condition and lag [*F*(2,22) = 6, *MSE* = 12.57, *p*<0.008] were revealed. Three Bonferroni-corrected paired-samples t-tests (*α = *0.017) affirmed that the T2-detection in the single condition differed only for lag 0 ms [*p*<0.003].

The average Delta E value obtained in the staircase procedure was 28.25. For Experiment 3b, performance in the single- and dual-task condition appeared to be similar at nonzero lags while showing a clear difference at lag 0 ms (see Figure 4D). The percentage correctly identified T2’s in the single-task condition averaged at 94.86%; for the dual-task condition this was 88.4% given correct T1 identification. A 2×3 ANOVA with the factors condition (single/dual task) and lag (0, 160 and 640 ms) was performed on the data shown in Figure 4D. An effect for condition [*F*(1, 11) = 47.98, *MSE* = 15.6, *p*<0.001] and lag [*F*(2, 22) = 20.93, *MSE* = 18.88, *p*<0.001] as well as an interaction effect between condition and lag [*F*(2, 22) = 11.54, *MSE* = 19.44, *p*<0.001] were found. Three Bonferroni-corrected paired-samples t-tests (*α* = 0.017) affirmed that the T2-detection in the single condition differed only for lag 0 ms [*p*<0.001].

The potential performance differences between single- and dual-task performance found in Experiments 1 (orientation) and 3a (motion), and Experiments 1 and 3b (color), were analyzed with two Bonferroni-corrected independent t-tests (*α* = 0.025). It appeared that the performance difference between single- and dual-task conditions for the 0 ms lag was smaller in Experiment 3a than in Experiment 1 [*t*(24) = 3.5, *p*<0.002]. This difference was also smaller in Experiment 3b than in Experiment 1 [*t*(24) = 2.43, *p*<0.023]. These results suggest that although there were no differences in performance for the letter task, participants were better at detecting a color-defined and motion-defined targets than an orientation-defined target in the dual-task condition.

## General Discussion

In this study we have investigated the costs associated with the processing of target features in a classic search task, using a dual-task paradigm. We measured detection accuracy for orientation, motion and color features in combination with an attentionally demanding RSVP task with varying temporal lags between targets. Previously, Joseph et al. [17] used a search task in which an orientation-defined feature had to be detected. When tested in isolation with increasing set sizes, this task exhibited a flat search slope, which suggests that (virtually) no attentional resources are required for detection. However, when combined with an RSVP task, a performance decrease for the orientation task was found for lags up to 400 ms between targets. This seminal result, which we replicate in Experiment 1, is remarkable because it conflicts with other dual-task studies that do not find interference between detection of an orientation stimulus and another concurrent task [47]–[50]. Given that Joseph et al.’s [17] study indicates that there are costs associated with the detection of a – supposedly preattentive – orientation-defined target, we were interested to know whether this also applies for other target-defining features. To elucidate this, we performed two experiments (Experiments 2a and 2b) in which the orientation-defined search target was replaced by a motion-defined and a color-defined target, respectively. In contrast to Experiment 1, both experiments showed unimpaired T2 performance in the dual-task conditions over all lags. These results may be explained by two complementary hypotheses.

The first is that they are caused by saliency differences. As shown by earlier research dual-task performance improves when targets with relatively high salience are used (e.g. [33], [34]). Additionally, processing salient features such as color and motion require less attentional capacities than processing e.g. letters [1]–[3], [21]. Hence, the second task in our dual task conditions might simply have been too easy to reveal task interference. Additionally, because of the very high T2 performance levels observed in our experiments, any interference could have been obscured by ceiling effects.

The second hypothesis is that the pattern we see in the results of Experiment 1 is based on feature-specific interference. The letter discrimination and the detection of an orientation-feature in Experiment 1 both require processing of orientation information. As both targets hold similar information they may have encountered limitations in feature-specific resources, resulting in performance decrease during simultaneous processing [32], [35]–[40]. Since motion and color detection require processing of information other than orientation, no such interference would be expected in Experiments 2a and 2b, which would explain the unimpaired dual-task performance. However, as noted above, the interpretation of the results of these experiments is hampered by the fact that T2 performance was close to ceiling.

In Experiment 3a and 3b motion and color were again studied but stimulus saliency was reduced by matching single-task performance to that observed in Experiment 1. This manipulation yielded interesting results: interference in the dual-task condition was reintroduced, but only at lag 0 ms. The fact that dual-task interference occurred also for these stimuli is at odds with traditional views stating that a “preattentive” search task should not suffer from an attention-demanding task carried out at the same time [35]–[39]. However, the findings are consistent with other research showing that attentional demands of the motion and color detection tasks are normally very low, but increase at lowered saliencies [1], [3], [31], [32]. It is important to note that, although some dual-task interference was observed in Experiment 3a and 3b, it was smaller than in Experiment 1 and absent for nonzero lags. As explained in the Introduction, many studies indicate that search tasks cannot be performed without attentional costs [9], [24], [26]–[28]. Such costs are expected to occur within the time window needed for attentional switching – say 100–200 ms [51]. The fact that the dual-task interference for the motion and color stimuli disappears already at lag 160 ms is therefore consistent with an explanation based on competition for attentional resources. In the dual-task condition of Experiment 1, a similar competition seems to take place, because there is reduced T2 performance at zero lag. The interference, however, extends well beyond 150 ms – up to at least 320 ms – which indicates that it is not only caused by attentional effects. In our opinion, the most likely cause for this late interference is a central processing bottleneck, resulting from the fact that similar features have to be processed for both the RSVP task and the (orientation) search task [35]–[40]. This later interference is reminiscent of the performance reduction occurring in the attentional blink (AB) paradigm, which is also attributed to a central processing bottleneck [52].

Summarizing, our results suggest that the large effect found in the Joseph et al.´s [17] study may well be the result of a worse-case situation, caused by competition for attentional resources as well as interference between simultaneous letter and orientation processing. Our results do, however, support their claim that basic feature search tasks cannot be performed purely preattentively, and without any cost, as was already suggested by other researchers [9], [17], [24], [26]–[28].

Lastly, an aspect of our findings that was not yet discussed is the pattern of T1 results across experiments. It is clear that performing a dual task may come at a cost for either T1 and/or T2 [3], [10], [31]. Our results are generally consistent with this notion: when T2 difficulty is increased, it is observed that T1 and/or T2 performance will suffer in the dual-task condition. However, the exact pattern of T1 results is not so easily explained. In particular the clear performance decrease in Experiment 3b is difficult to understand, as previous research found that in particular color detection can be combined with additional tasks at no detectable cost [1], [3], [31]. Future research should, therefore, focus on the interaction between different types of primary and secondary tasks, including, for example, conditions in which the order of RSVP and search task are reversed.

### Conclusions

Concluding, it appears that search for targets with a unique feature indeed comes at a cost. One way of revealing this is by requiring subjects to perform the search task simultaneously with another highly attention-demanding task. Depending on the stimuli used in the search task, the cost may be substantial, small, or even negligible. We explain this by a combination of effects of salience, competition for attentional resources and competition for processing resources. Thus, if you are trying to catch the attention of a friend in a crowd, and your friend is doing something else at the same time, you may have trouble being noticed, but it will certainly help to wave your arm or wear distinct colors.

## Supporting Information

Table S1 **Representation of RGB values and luminance for each color.**
(DOCX)
