# Supplementary material for: Attentional Requirements on Feature Search Are Modulated by Stimulus Properties
Source: PLoS One. 2013 Jan 7;8(1):e53093. doi: 10.1371/journal.pone.0053093 (PMC3538745; doi:10.1371/journal.pone.0053093)
Supplement: Table S1 — Representation of RGB values and luminance for each color. (DOCX) [file pone.0053093.s001.docx]

Table 1. Representation of RGB values for each target and distractor color.

| **Color** | **RGB Value** | **Luminance in cd/m^2^** |
| --- | --- | --- |
| color 1 | 255,0,0 | 17,01112 |
| color 2 | 254,9,0 | 17,01605 |
| color 3 | 253,16,0 | 17,00563 |
| color 4 | 252,22,0 | 17,01843 |
| color 5 | 251,26,0 | 17,0014 |
| color 6 | 250,30,0 | 17,00498 |
| color 7 | 249,34,0 | 17,02995 |
| color 8 | 248,36,0 | 16,97745 |
| color 9 | 247,39,0 | 16,98301 |
| color 10 | 246,42,0 | 17,00183 |
| color 11 | 245,45,0 | 17,03418 |
| color 12 | 244,47,0 | 17,01565 |
| color 13 | 243,49,0 | 17,00377 |
| color 14 | 242,51,0 | 16,99861 |
| color 15 | 241,53,0 | 17,00024 |
| color 16 | 240,55,0 | 17,00874 |
| color 17 | 239,57,0 | 17,02417 |
| color 18 | 238,58,0 | 16,96517 |
| color 19 | 237,60,0 | 16,99152 |
| color 20 | 236,62,0 | 17,02499 |
| color 21 | 235,63,0 | 16,97621 |
| color 22 | 234,65,0 | 17,02085 |
| color 23 | 233,66,0 | 16,97846 |
| color 24 | 232,67,0 | 16,93847 |
| color 25 | 231,69,0 | 16,99848 |
| color 26 | 230,71,0 | 17,06589 |
| color 27 | 229,72,0 | 17,03648 |
| color 28 | 228,73,0 | 17,00951 |
| color 29 | 227,74,0 | 16,98499 |
| color 30 | 226,75,0 | 16,96292 |
| color 31 | 225,76,0 | 16,94332 |
| color 32 | 224,78,0 | 17,03899 |
| color 33 | 223,79,0 | 17,02606 |
| color 34 | 222,80,0 | 17,01561 |
| color 35 | 221,81,0 | 17,00766 |
| color 36 | 220,82,0 | 17,00221 |
| color 37 | 219,83,0 | 16,99926 |
| color 38 | 218,84,0 | 16,99882 |
| color 39 | 217,85,0 | 17,00091 |
| color 40 | 216,86,0 | 17,00551 |
| color 41 | 215,87,0 | 17,01265 |
| color 42 | 214,88,0 | 17,02232 |
| color 43 | 213,89,0 | 17,03454 |
| color 44 | 212,90,0 | 17,0493 |
| color 45 | 211,91,0 | 17,06661 |
| color 46 | 210,92,0 | 17,08649 |
